# Total or Near-total Thyroidectomy in treatment of Thyroid Cancer

**DOI:** 10.12669/pjms.38.6.5765

**Published:** 2022

**Authors:** Gang Li, Lihong Wu

**Affiliations:** 1 Gang Li, Department of General Surgery, The First Affiliated Hospital of Qiqihar Medical College, Qiqihar 161041, Heilongjiang, China; 2 Lihong Wu Department of General Surgery, The First Affiliated Hospital of Qiqihar Medical College, Qiqihar 161041, Heilongjiang, China

**Keywords:** Thyroid cancer, Total thyroidectomy, Near-total thyroidectomy, Clinical efficacy

## Abstract

**Objectives:**

To observe the clinical efficacy on total or near-total thyroidectomy in the treatment of thyroid cancer.

**Methods:**

Ninety-four patients with thyroid cancer treated in Meiris Branch of the First Affiliated Hospital of Qiqihar Medical College from June 2018 to June 2020 were selected as subjects. According to different surgical methods, they were divided into observation group and control group, with 47 patients in each group. The control group was treated with total thyroidectomy, while the observation group received near-total thyroidectomy. The two groups were both followed up for one year. The therapeutic effect, surgery-related indexes (surgical duration, intraoperative bleeding volume, postoperative analgesia time and postoperative 24-h VAS score), the incidence of complications three months after surgery, and the serum levels of relevant indexes [parathyroid hormone (PTH), calcium ion (Ca^2+^) and signal transducer and activator of transcription 3 (STAT3)] before and 14 d after surgery were compared between the two groups. The difference in quality of life [the European Organization for Research and Treatment of Cancer Quality of Life Questionnaire (EORTC QLQ-C30)] between the two groups one year after surgery was observed.

**Results:**

During the one year follow-up, there was no death in both groups. The total efficacy of the observation group was higher than that of the control group (0.05). The surgical duration, postoperative analgesia time and postoperative 24-h VAS score of the observation group were higher than those of the control group (*p*<0.05). However, no statistically significant difference was found in intraoperative bleeding volume between the two groups (*p>* 0.05). Three months after surgery, the total incidence of postoperative complications in the observation group was higher than that in the control group (*p<* 0.05). Fourteen days after surgery, the levels of PTH, Ca^2+^ and STAT3 in the two groups were lower than those before surgery, and the levels in the observation group were lower than those in the control group (*p<* 0.05). One year after surgery, cognitive, emotional, role, social and physical scores in the observation group were all lower than those in the control group, without statistically significant differences (*p>* 0.05).

**Conclusion:**

Total thyroidectomy is effective in the treatment of thyroid cancer, but has many postoperative complications. Clinicians need to choose the appropriate surgical method according to the actual condition of patients.

## INTRODUCTION

Thyroid cancer is a common malignant tumor in clinic. Relevant study has pointed out that the incidence of thyroid cancer is increasing significantly with the application of thyroid color Doppler ultrasonography in physical examination, and higher in females than males. It has become one of the diseases threatening the health of residents in China.^1^ At present, surgical resection is the first choice for clinical treatment, but the anatomical position of the thyroid is complex and there are many clinical surgical problems.^2^ Total and near-total thyroidectomy are two commonly used surgical methods in clinic. However, some researchers have pointed out that the rate of disease recurrence at the late stage of near-total resection is high, and secondary surgery will bring great pain and psychological pressure to patients.^3^ On this basis, this study mainly observed the therapeutic effects of total and near-total thyroidectomy, so as to provide more reference for clinicians to choose treatment plans.

## METHODS

Ninety-four patients with thyroid cancer treated in Meiris Branch of the First Affiliated Hospital of Qiqihar Medical College from June 2018 to June 2020 were selected as subjects. According to different surgical methods, they were divided into observation group and control group, with 47 patients in each group. No significant differences were found in clinical general data such as gender, age, course of disease, body mass index (BMI) and clinical stage between the two groups (*p>* 0.05), suggesting comparability. Table-I.

**Table-I:** Comparison of clinical general data between the two groups [*x̅*± *s,* n (%)].

Group	n	Gender		Age (years)	Course of disease (year)	BMI (kg/m2)	Clinical stage		
	
		Male	Female				I stage	II stage	III stage
Observation group	47	16 (34.04)	31 (65.96)	42.31 ± 6.59	1.23 ± 0.56	20.35 ± 0.94	28 (59.58)	14 (29.78)	5 (10.64)
Control group	47	13 (27.66)	34 (72.34)	43.15 ± 6.43	1.30 ± 0.49	20.41 ± 0.87	26 (55.32)	15 (31.91)	6 (12.77)
t or Χ^2^	0.449			0.625	0.645	0.321	0.437		
P	0.503			0.533	0.521	0.748	0.662		

### Ethical approval

The study was approved by the Institutional Ethics Committee of Meris Branch of the First Affiliated Hospital of Qiqihar Medical College on March 12, 2020 (No.: 2020008), and written informed consent was obtained from all participants.

### Inclusion criteria:

• Patients meeting the diagnostic criteria of thyroid cancer in the Guidelines for Diagnosis and Treatment of Thyroid Nodules and Differentiated Thyroid Cancer^4^;
• Patients with estimated survival > 1 year;
• Patients who were informed of this study and signed the informed consent.

### Exclusion Criteria:

• Patients with diseases of the heart, liver, kidney or other important organs, or combined with other malignant tumors;
• Patients with coagulation dysfunction;
• Patients with undifferentiated thyroid cancer;
• Patients with a previous history of neck surgery, or adhesion between cervical great vessels, tracheae and thyroid cancer.

After admission, relevant preoperative examinations were improved, and all the patients lied supine without pillow and exercised head and neck hypsokinesis and hyperextension.

### Observation group

The patients were in the supine position, with the neck and chest fully exposed and routinely disinfected. After tracheal intubation and general anesthesia, a 4-6 cm incision was made at two transverse fingers above the sternum to fully expose the thyroid. Then, the suspensory ligament of thyroid was separated, the superior pole vessels of the thyroid were ligated, and the middle and lower veins were ligated and cut off to carefully identify the parathyroid gland and recurrent laryngeal nerve. On the basis of protecting the recurrent laryngeal nerve, the thyroid capsule and thin gland at the entrance of the recurrent laryngeal nerve were preserved, and most gland was resected and removed along the thyroid capsule from the bottom to the top. After hemostasis, suturing, indwelling and drainage, routine dressing was performed.

### Control Group

The surgical method was the same as that of the observation group. Only most thyroid was resected, and the dorsal gland and capsule of the thyroid were preserved.

### Index Detection

Fasting venous blood was collected before and 14 days after surgery. Signal transducer and activator of transcription 3 (STAT3) was detected using chemiluminescence immunoassay. Parathyroid hormone (PTH) and calcium ion (Ca^2+^) levels were determined by ELISA.

### Evaluation Criteria

Therapeutic effect^5^ Remarkable: the clinical symptoms of the patients disappeared and all indexes returned to normal; effective: the clinical symptoms of the patients were improved and all indexes recovered; invalid: the clinical symptoms of the patients did not change or even aggravated, and various indexes were abnormal. Total effective rate = markedly effective rate + effective rate.Pain of the patients was evaluated using the visual analogue scale (VAS)^6^, scored 1~10. The higher the score, the severer the pain.The quality of life was evaluated using the European Organization for Research and Treatment of Cancer Quality of Life Questionnaire (EORTC QLQ-C30)^7^, including cognitive, emotional, role, social and physical fields. The higher the score, the better the patients’ quality of life.The therapeutic effect, surgery-related indexes (surgical duration, intraoperative bleeding volume, postoperative analgesia time and postoperative 24-h VAS score), the incidence of complications (recurrent laryngeal nerve injury, hoarseness, hypocalcemia and dyspnea), the serum levels of relevant indexes (PTH, Ca^2+^ and STAT3) before and 14 days after surgery, and quality of life (EORTC QLQ-C30) were compared between the two groups.

### Statistical Analysis

The data were analyzed using SPSS18.0. The measurement data were expressed as mean ± standard deviation, and those with normal distribution were analyzed using the t test. The enumeration data were analyzed using the *χ^2^* test. The ranked data were analyzed by the rank-sum test. *P<* 0.05 was considered as statistically significant.

## RESULTS

During the one year follow-up, there was no death in both groups. The total effective rate of the observation group was higher than that of the control group (*p<* 0.05). Table-II.The surgical duration, postoperative analgesia time and postoperative 24-hour VAS score of the observation group were higher than those of the control group (*p<* 0.05). However, no statistically significant difference was found in intraoperative bleeding volume between the two groups (*p>* 0.05). Table-III.

**Table-II:** Comparison of therapeutic effect between the two groups [n (%)].

Group	n	Remarkable	Effective	Invalid	Total effective rate
Observation group	47	35 (74.47)	11 (23.40)	1 (2.13)	46 (97.87)
Control group	47	27 (57.45)	13 (27.66)	7 (14.89)	40 (85.11)
z/x^2^		1.990		4.919	
P		0.047		0.027	

**Table-III:** Comparison of surgery-related indexes between the two groups ( (*x̅* ± *s*).

Group	n	Surgical duration (min)	Intraoperative bleeding volume (mL)	Postoperative analgesia time (h)	Postoperative 24-h VAS score
Observation group	47	81.25 ± 13.16	54.12 ± 8.78	21.43 ± 3.95	5.12 ± 1.13
Control group	47	73.58 ± 11.37	52.51 ± 8.34	15.63 ± 3.84	4.45 ± 1.07
t		3.023	0.911	7.218	2.952
P		0.003	0.364	0.000	0.004

Three months after surgery, the total incidence of postoperative complications in the observation group was higher than that in the control group (*p<* 0.05).Table-IV.

**Table-IV:** Comparison of postoperative complications between the two groups [n (%)]

Group	n	Recurrent laryngeal nerve injury	Hoarseness	Hypocalcemia	Dyspnea	Total incidence
Observation group	47	5 (10.64)	6 (12.77)	2 (4.26)	2 (4.26)	15 (31.91)
Control group	47	1 (2.13)	2 (4.26)	1 (2.13)	1 (2.13)	5 (10.64)
x^2^	-					6.351
P	-					0.012

Fourteen d after surgery, the levels of PTH, Ca^2+^ and STAT3 in the two groups were lower than those before surgery, and the levels in the observation group were lower than those in the control group (*p<* 0.05).Table-V. One year after surgery, cognitive, emotional, role, social and physical scores in the observation group were all lower than those in the control group, without statistically significant differences (*p>* 0.05). Table-VI.

**Table-V:** Comparison of serum relevant indexes between the two groups ( (*x̅* ± *s*).

Group	n	Time	PTH (pg/mL)	Ca^2+^(mmol/L)	STAT3 (pg/mL)
Observation group	47	Before surgery	83.56 ± 8.33	2.24 ± 0.31	116.53 ± 6.59
		14 d after surgery	43.59 ± 5.67 ^*#^	1.82 ± 0.21^*#^	38.76 ± 5.12^*#^
Control group	47	Before surgery	84.12 ± 8.41	2.23 ± 0.32	114.75 ± 6.62
		14 d after surgery	51.42 ± 6.03 ^*^	1.97 ± 0.22^*^	65.58 ± 6.13^*^

*Notes:* Compared with before surgery,

* p< 0.05; compared with 14 d after surgery in the control group, p< 0.05.

**Table-VI:** Comparison of quality of life between the two groups ( (*x̅* ± *s*).

Group	n	Cognition	Emotion	Role	Society	Physical
Observation group	47	66.53 ± 8.17	70.53 ± 7.94	68.59 ± 8.13	71.33 ± 8.84	68.85 ± 7.76
	47	68.32 ± 8.59	71.42 ± 7.88	69.41 ± 8.89	72.95 ± 8.58	69.54 ± 7.73
t		1.035	0.545	0.467	0.902	0.432
P		0.303	0.587	0.642	0.370	0.667

## DISCUSSION

Thyroid cancer is a common head and neck malignant tumor in clinic, which is the 5th largest malignant tumor threatening women’s health. Most patients have no special feelings after onset, and may present symptoms such as hoarseness and dysphagia in the later stage.^8^ In recent years, with the change in people’s lifestyle, the incidence of thyroid cancer is increasing year by year, which can cause a great impact on the quality of life of patients. Without timely treatment, it can threaten the life safety of patients.^9^ Clinically, it is considered that surgical resection is the preferred treatment, among which total and near-total thyroidectomy are the most widely used.

Some researchers have pointed out that the initial therapeutic effect of patients with thyroid cancer can directly affect their postoperative quality of life and survival rate. For the initial treatment, total thyroidectomy results in better prognosis.^10^ In this study, our results showed that the clinical therapeutic effect of the observation group was better than that of the control group. It may be caused by total thyroidectomy that can remove the primary lesion to the greatest extent and improve the clinical therapeutic effect.^11^ In addition, our study also revealed that the surgical duration of the observation group was shorter than that of the control group, and the postoperative analgesia time and 24-h VAS score were higher than those of the control group, which are similar to the results of Chien-Ling Hung et al.^12^

These outcomes may be because:: It is needed to separate and retain certain gland tissue during near-total thyroidectomy, which can prolong the surgical duration; Moreover, total thyroidectomy is more traumatic, and can increase the probability of damage to tissues and organs around the thyroid, increase the degree of postoperative pain and prolong the postoperative analgesia time.

Another study has shown that total thyroidectomy can reduce the injury rate of the parathyroid gland, cause loss of thyroid function and increase the incidence of complications.^13^ Among them, hypocalcemia is one of the most common complications. Mild patients can present numbness and convulsions of hands and feet, and severe patients may suffer from muscle spasm, cognitive impairment and even dyspnea, endangering life safety.^14^ The results of this study showed that the incidence of postoperative complications in the observation group was higher than that in the control group, which is similar with the results of Marcin Barczyński et al.^15^ The reason is that during total thyroidectomy, no protective measures were taken for the parathyroid gland and recurrent laryngeal nerve in the posterior capsule of the thyroid, which destroyed the blood supply of the parathyroid gland and microvessels, increased recurrent laryngeal nerve injury, and enhanced the incidence of postoperative complications such as hoarseness and dyspnea. In addition, the destruction of the parathyroid gland and its microvessels can lead to the decline in parathyroid function, low calcium and numbness of hands and feet.^16^

Parathyroid gland is an important endocrine gland in the human body mainly secreting PTH, which can act on the bone and kidney and regulate serum calcium level.^17^-^19^ It has been pointed out that PTH can effectively reflect the thyroid function of patients,^20^ and thyroidectomy can lead to potential insufficient PTH secretion after surgery.^21^ Poupak Fallahi et al.^22^ Also found that PTH detection after thyroid surgery can well predict hypocalcemia. In this study, it was found that PTH and Ca^2+^ levels in the observation group were lower than those in the control group.

### The reasons may include

The parathyroid gland is located in the thyroid gland. When total thyroidectomy, it is easy to cut the parathyroid gland by mistake, resulting in insufficient parathyroid secretion. During total thyroidectomy, parathyroid injury may be caused by suturing, ligation, traction, clamping, electrocoagulation and other accidents, leading to ischemia or even necrosis, and parathyroidism. STAT3 can regulate a variety of cytokines, growth factors and angiogenesis factors. It plays an important role in gene transcription and expression, and is closely related to the occurrence, development and prognosis of tumors. It is an index for predicting the prognosis of a variety of tumors in recent years.^23^,^24^ Another study has shown that the signal transduction pathway is activated and transferred from the cytoplasm to the nucleus, which plays an important role in regulating and inducing the expressions of multiple genes and anti-tumor cell apoptosis.^25^ In our study, the STAT3 level of the observation group was lower than that of the control group, suggesting that total thyroidectomy can improve the prognosis of the patients.

With the formation of modern patient-oriented medical concept, the core of surgical treatment has gradually changed from “disease” to “patient”. Based on improving the safety of surgery and perioperative links as well as enhancing clinical efficacy, we also need to pay attention to the changes in patients’ quality of life. The results of this study showed that the scores of all dimensions in EORTC QLQ-30 in the observation group were lower than those in the control group. Although there was no statistically significant difference between the two groups, it also suggests to a certain extent that near-total thyroidectomy will not reduce the clinical effect of patients, which is similar to the results of Ladurner et al.^19^ This may be related to the low incidence of postoperative complications of total thyroidectomy.

### Limitations of the study

The number of subjects included in this study was limited, so the conclusions drawn may not be very convincing.We only analyzed and discussed the cases included in our hospital, which may not be representative enough. We look forward to a multi-center study in the future to reach more comprehensive conclusions.

## CONCLUSION

Total thyroidectomy is effective in the treatment of thyroid cancer, but has long surgical duration, severe postoperative pain and many postoperative complications. Clinicians need to choose the appropriate surgical method according to the actual condition of patients.

### Authors’ Contributions:

**GL & LW:** Designed this study, prepared this manuscript, are responsible, accountable for the accuracy and integrity of the work.

**LW:** Collected and analyzed clinical data.

**GL:** Significantly revised this manuscript.
